# Polyprenyl immunostimulant treatment during experimental feline herpesvirus-1 infection is associated with reduced ocular disease after conjunctival rechallenge

**DOI:** 10.3389/fvets.2026.1834777

**Published:** 2026-06-08

**Authors:** R. Eric Heidel, Tanya Kuritz, Roger K. Maes, Danielle Thompson, Morgan Beasley, Craig R. Reinemeyer

**Affiliations:** 1 Department of Surgery, College of Medicine, University of Tennessee Health Science Center, Knoxville, TN, United States; 2 Sass & Sass Inc., Oak Ridge, TN, United States; 3 Veterinary Diagnostic Laboratory, College of Veterinary Medicine, Michigan State University, East Lansing, MI, United States; 4 East Tennessee Clinical Research Inc., Rockwood, TN, United States

**Keywords:** controlled clinical trial, FeHV-1, feline, feline ocular disease, innate immunity, ocular challenge, polyprenyl immunostimulant

## Abstract

Polyprenyl immunostimulant (PI) was, at the time of this study, a USDA-licensed veterinary biologic for the treatment of feline rhinotracheitis. In two previous controlled challenge trials, PI administration reduced the severity of acute feline rhinotracheitis in specific pathogen-free (SPF) cats (1). We replicated the oral PI challenge-treatment protocol to evaluate humoral and virologic outcomes, measured by serum virus-neutralizing antibody titers and virus isolation, and to assess clinical ocular disease after conjunctival rechallenge. During the study, the frequency of virus isolation and the virus-neutralizing antibody (VN) titers did not vary significantly between the treatment and placebo groups. After the rechallenge, all placebo controls (8/8) developed ocular disease, whereas 10/16 PI-treated cats developed conjunctivitis/ocular disease, and 6/16 remained asymptomatic. PI treatment was associated with a 37.5% reduction in the incidence of ocular disease after conjunctival rechallenge. We hypothesize that PI may reduce the likelihood of clinical ocular disease after FeHV-1 rechallenge by priming innate cell-mediated immune responses.

## 1 Introduction

Feline herpesvirus-1 (FeHV-1) causes acute upper respiratory and ocular disease and can establish latency with intermittent recrudescence and viral shedding; after experimental exposure, a lag phase of several days precedes peak shedding and clinical signs (1–4). Disease severity varies from mild upper respiratory or ocular signs to clinically significant keratitis, ulceration, and chronic or recurrent ocular disease, with greater concern in young, stressed, crowded, or otherwise susceptible cats. Like other alphaherpesviruses, FeHV-1 is characterized by latency and reactivation, although the clinical syndrome and host range are feline-specific (2–5). Legendre et al. reported that polyprenyl immunostimulant (PI) reduced the severity of acute feline rhinotracheitis in SPF cats during the acute phase after experimental FeHV-1 challenge (1).

This study aimed to assess the effects of PI treatment on humoral/virologic outcomes and clinical effects after ocular rechallenge, as measured by virus isolation assay and virus-neutralizing (VN) titers, and to evaluate disease incidence. In Phase 1 (0–44 days post challenge [DPC]), we replicated the challenge protocol (1) and followed cats until complete clinical sign resolution without antibiotic intervention. General health observations continued through the interval before Phase 2 rechallenge.

In Phase 2, we assessed whether PI treatment was associated with reduced incidence of ocular disease in the qualified rechallenge cohort; the cats were rechallenged via conjunctival route with the same FeHV-1 strain at 62 DPC and followed through post-rechallenge observation and serology/virus isolation testing at 87 DPC; group allocations were unmasked at 102 DPC.

We report that treatment with PI for 0–14 DPC during the first challenge was associated with reduced ocular disease after the rechallenge. The VN titers did not appear to be associated with the rechallenge outcome.

We hypothesize that PI may reduce the likelihood of clinical ocular disease after FeHV-1 rechallenge by priming innate cell-mediated immune responses.

## 2 Materials and methods

### 2.1 Study design

This placebo-controlled clinical study was designed and conducted to meet USDA requirements under 9 CFR 113.209 and USDA APHIS Center for Veterinary Biologics Memorandum 800.202 (6). The animal research was conducted in 2023 by East Tennessee Clinical Research, Inc. (ETCR), in Rockwood, TN, in compliance with a protocol approved by the facility’s Institutional Animal Care and Use Committee (ETCR-23-0308). All cats remained asymptomatic after the study and were placed for adoption.

**Figure 1:**
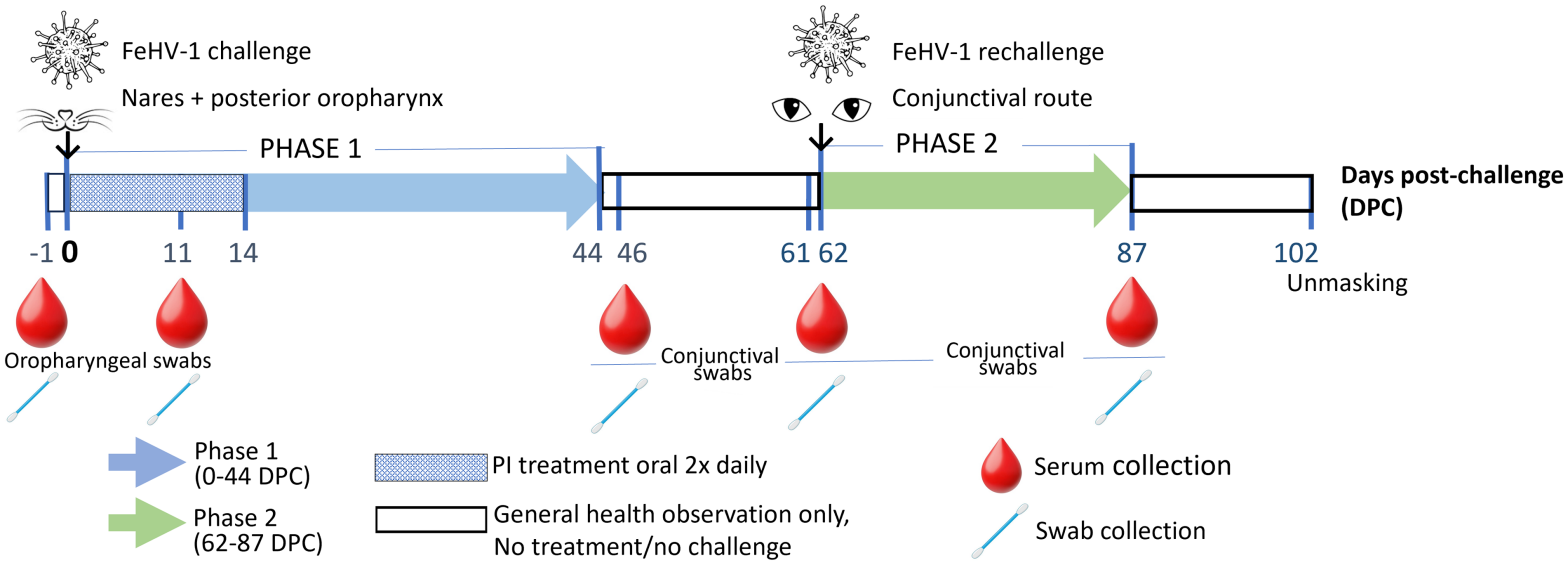
Study flowchart. Phase 1 included nasopharyngeal challenge with FeHV-1, oral PI or placebo treatment twice daily for 14 DPC, and follow-up until clinical signs resolved at 44 DPC. Phase 2 began with conjunctival FeHV-1 rechallenge on 62 DPC and continued through post-rechallenge follow-up to 87 DPC, when clinical signs resolved. Blood-drop icons indicate serum collection; swab icons indicate protocol-specified swab collection, including oropharyngeal swabs during Phase 1 and conjunctival swabs before and after Phase 2 rechallenge. Group allocations were unmasked at 102 DPC. DPC, days post-challenge; PI, polyprenyl immunostimulant.

The study flowchart is shown in Figure 1. The time was counted from the day of the first challenge (Day 0) through the sign resolution in all cats. The study timeline was divided into Phase 1 (P1: 0–44 DPC, challenge and treatment) and Phase 2 (P2: DPC 62–87, ocular rechallenge). The initial challenge was administered to the upper respiratory tract, including the nares and posterior oropharynx; the rechallenge was administered bilaterally into the conjunctival sacs. Both phases were followed by non-study days during which general health observations were conducted. The study was terminated and unmasked on 102 DPC.

### 2.2 Materials

The FeHV-1 (FVR-SGE) virus strain was kindly provided by Dr. Michael Lappin of Colorado State University. Polyprenyl immunostimulant was supplied by VetImmune LLC, Oak Ridge, TN.

### 2.3 Phase 1: challenge and treatment

Thirty specific pathogen-free (SPF), unvaccinated cats, 7-8 weeks of age (Marshall Bioresources), were randomized 2:1 (PI *n* = 20; placebo control *n* = 10). The experimental design was essentially the same as in the clinical trials of 2007 and 2013 described by Legendre et al. (1). On Day 0, all 30 cats received 1 mL of FeHV-1 FVR-SGE at 10^6^ TCID50/mL, administered approximately 0.33 mL per nostril and 0.33 mL to the posterior oropharynx. The cats were followed by trained, masked personnel until complete resolution of the disease, without antibiotic intervention, by Day 44 post-challenge (DPC).

### 2.4 Phase 2: conjunctival rechallenge (P2)

For the rechallenge, we selected a cohort of subjects (24/30) with VN titers <128 and negative on virus isolation on 46 DPC to limit the confounding effects of potential shedding or very high antibody levels. The group assignments made in P1 remained masked until the end of the study.

On 62 DPC, all 24 cats received 30 μL of 2.6 × 10^7^ TCID50/mL of FeHV-1 strain FVR-SGE into the conjunctival sacs of both eyes with gentle rubbing. Daily ocular observations and scoring were performed by trained masked personnel. Phase 2 ended when cats resolved ocular signs by 87 DPC.

General health observations were conducted until 102 DPC, when the trial was terminated and data unmasked.

### 2.5 Laboratory testing

The FeHV-1 FVR-SGE challenge virus was propagated, titrated, and tested by the Virology Section of the Michigan State University Veterinary Diagnostic Laboratory (MSU VDL), an AAVLD-accredited full-service animal diagnostic laboratory. Viral stock preparation was performed in Crandell-Reese feline kidney (CRFK) cells in fetal bovine serum-containing medium (FBS C/G) supplemented with ciprofloxacin and gentamicin (MSU VDL SOP.VIRO.39.5).

Virus isolation was performed on swabs processed in transport liquid, filtered through 0.45 μm filters, and inoculated onto CRFK cell cultures incubated in FBS C/G medium. Virus-induced cytopathic effect (CPE) was reported as 1 (virus detected) or 0 (no virus detected) based on the presence or absence of CPE after culture observation and laboratory review (MSU VDL SOP.VIRO.56.1).

Serum FeHV-1 virus-neutralizing antibody titers were measured by the FeHV-1-neutralization test. Briefly, heat-inactivated serum samples were serially diluted and incubated with a fixed amount of FeHV-1 virus, followed by the addition of CRFK indicator cells. After incubation, wells were evaluated microscopically for CPE. The VN titer was reported as the reciprocal of the highest serum dilution that completely neutralized virus-induced CPE. Each run included cell controls, virus back-titration, positive and negative serum controls, and laboratory review before reporting (MSU VDL SOP.VIRO.38.7).

### 2.6 Clinical scoring

The scoring system was binary as preferred by the regulatory authority (6): the presence of any or a combination of the disease signs was assigned a score of 1, while cats with unaffected eyes received a score of 0 on that day. The scoring system was reviewed by the USDA and satisfied requirements for the primary outcome. Ocular disease was defined as conjunctivitis, blepharitis, or keratitis of any severity or a combination of any of those in one or both eyes. All cats had resolved ocular disease on 87 DPC without interventions. Serology was followed through 87 DPC.

### 2.7 Statistical analysis and validation

The primary Phase 2 ocular disease outcome was analyzed as a relative-risk comparison between the PI-treated and placebo groups. Relative risk, absolute risk reduction, number needed to treat, 95% confidence intervals, and the associated *p*-value were calculated using the MedCalc Relative Risk calculator, with Sheskin (7) as the statistical reference. VN titers were compared between groups using the Mann–Whitney *U* test. Statistical significance was assumed at a two-sided alpha value of 0.05, and all analyses were performed using IBM SPSS Statistics (Version 31).

## 3 Results

### 3.1 Virus isolation and neutralizing antibody titers

All 30 cats were negative for FeHV-1 on −1 DPC (before P1, the challenge-treatment phase). At 11 DPC, virus isolation was positive in 4/19 PI-treated (the sample was not collected from one cat) and 1/10 placebo cats; one PI-treated cat had positive virus isolation on 11 and 46 DPC. All preceding and later sampling points were negative.

Available sera were seronegative before challenge; VN titers developed after challenge, with all available 46 DPC samples showing measurable titers. The study data are summarized in Table 1. There were no significant differences in the FeHV-1 VN antibody titers between the treatment and control arms at Time −1 DPC (*p* = 1.00), 11 DPC (*p* = 0.61), 46 DPC (*p* = 0.67), 61 DPC (*p* = 0.44), or 87 DPC (*p* = 0.75). The last sampling point at 87 DPC included 24 cats that received ocular rechallenge.

**Table 1:** FeHV-1-neutralizing antibody titers in the cats during the study and post-rechallenge outcome.

CatID	Treatment group	Sampling points, DPC					Diseased post-rechallenge (Y or N)
		-1	11	46	61	87	
The placebo-treated cats all diseased after conjunctival rechallenge							
M234023	Placebo	<4	<4	32	16	256	Y
M234236	Placebo	<4	4	32	32	>4,096	Y
M234261	Placebo	<4	16	16	32	256	Y
M234295	Placebo	<4	<4	16	32	128	Y
M234512	Placebo	<4	8	32	16	512	Y
M234554	Placebo	<4	4	64	64	128	Y
M234121	Placebo	<4	8	32	32	2048	Y
M234155	Placebo	ND	4	16	32	256	Y
The PI-treated cats not diseased after conjunctival rechallenge							
M234058	Treated	<4	4	16	128	256	N
M234007	Treated	<4	4	16	512	128	N
M234104	Treated	<4	<4	32	32	256	N
M234147	Treated	<4	<4	32	32	512	N
M234244	Treated	<4	8	64	64	128	N
M234490	Treated	<4	8	32	1,024	512	N
The PI-treated cats diseased after conjunctival rechallenge							
M234252	Treated	<4	<4	8	8	256	Y
M234180	Treated	<4	8	32	128	256	Y
M234015	Treated	<4	<4	32	32	128	Y
M234279	Treated	<4	4	16	16	512	Y
M234431	Treated	<4	<4	32	32	256	Y
M234449	Treated	ND	4	16	8	256	Y
M234465	Treated	<4	4	8	16	2048	Y
M234482	Treated	<4	8	16	4	1,024	Y
M234520	Treated	<4	8	32	32	1,024	Y
M234546	Treated	<4	4	64	64	256	Y
Cats not included into conjunctival rechallenge							
M234287	Placebo	<4	8	128	128	NI	NI
M234538	Placebo	<4	<4	ND	512	NI	NI
M234040	Treated	<4	8	512	128	NI	NI
M234074	Treated	<4	4	256	2048	NI	NI
M234082	Treated	<4	4	128	64	NI	NI
M234473	Treated	<4	8	256	128	NI	NI

NI, not included in the P2 rechallenge trial. High-titer samples from cats M234121, M234236, and M234482 were retested to validate assay reproducibility.

### 3.2 Ocular disease incidence after ocular rechallenge

The 24/30 cats enrolled in the P2 rechallenge had negative virus isolation and VN titers <128 at 46 DPC.

All cats in the placebo control arm (8/8, 100%) and 10/16 cats in the PI-treated arm (62.5%) developed ocular disease limited to conjunctivitis; six PI-treated cats remained asymptomatic throughout the trial (Table 2). Relative-risk analysis using the MedCalc calculator showed an absolute risk reduction of 37.5% (95% CI 8.6–57.2%), with an NNT of 2.67 (95% CI 1.41–25.30; *p* = 0.015).

**Table 2:** Ocular disease incidence in P2, post-rechallenge (*n* = 24).

Study group	N	Disease		Risk*
		Y	N	
Placebo	8	8	0	1.000
Treated	16	10	6	0.625

*Risk = proportion of cats in each study group that developed ocular disease after the P2 conjunctival rechallenge. Absolute risk reduction = 37.5% (95% CI 8.6–57.2%); number needed to treat = 2.67 (95% CI 1.41–25.30). All values calculated using MedCalc Relative Risk calculator.

## 4 Discussion

In this controlled FeHV-1 challenge study, PI administration was not associated with detectable differences in virus isolation or serum VN titers, but treated cats had a lower incidence of ocular disease after ocular rechallenge. These findings suggest that the observed clinical effect was not explained by the measured humoral response. The disease dynamic was essentially as previously reported (1–3, 5). After the first challenge and treatment, most cats in both groups developed disease of varying severity in the acute phase during 0–15 days post-challenge (DPC), after which several cats experienced intermittent mild signs, consistent with published reports (1, 2, 5). The VN antibody titers by 46 DPC were similar to reports from various vaccine efficacy trials: a modest increase in VN titers, generally not exceeding 128, after 40–81 days post-vaccination in both study arms (3, 4, 8). Virus isolation was the virologic assay; we did not evaluate molecular shedding.

After ocular rechallenge of the 24 qualified cats, all (*n* = 8, 100%) in the placebo arm and 10/16 (62.5%) in the treatment arm developed ocular disease. The treatment with PI was therefore associated with a 37.5% absolute reduction in the incidence of ocular disease. The ocular signs in cats were limited to conjunctivitis of different severity; no blepharitis or keratitis was noted. No formal vision testing was performed. The VN antibody titers overlapped, and there was no significant difference in the titers between the groups pre- and post-rechallenge.

In Phase 2, the ocular rechallenge analysis was performed in a qualified cohort of 24/30 originally randomized cats rather than as a full intention-to-treat analysis. Although Phase 1 treatment allocation remained masked when Phase 2 eligibility was determined, post-randomization eligibility selection may introduce bias and limit causal interpretation. The Phase 2 findings should therefore be interpreted as a qualified rechallenge cohort result. In addition, the primary ocular endpoint was binary and did not capture disease severity or duration.

The highest VN titers (1,024 to >4,096) were detected on 87 DPC in affected cats (3/10 in the treatment and 2/8 in the placebo control groups). The VN measurements in the sera of the cats in the placebo (M234121 and M234236) and treatment (M234482) groups were retested to confirm the assay reproducibility at those high titers. At the same time, the 6 treated cats that were clinically unaffected by the rechallenge had VN titers ranging from 128 to 512. These observations are preliminary due to the study’s small sample size, and VN titers are considered descriptive humoral markers of protection.

Cellular responses are considered central to the control of FHV-1, whereas serum antibody levels have limited value for predicting individual protection or disease risk (9–12). Because FHV-1 infection is primarily localized rather than viremic, circulating VN titers may not fully reflect the immune mechanisms most relevant to protection at ocular and upper respiratory mucosal sites. Thus, the absence of a detectable VN difference between groups does not exclude a treatment-associated biologic effect. Wu et al. recently reported that protection conferred by the FeHV-1 modified live vaccine was associated with cellular immune responses involving Th1-mediated mechanisms (8).

This is not unexpected because PI is an immune modulator shown to upregulate the expression of CD11b, a monocyte/macrophage activation marker, on the peripheral blood mononuclear cells (PBMC) of the cats, both in the FeHV-1 controlled challenge study and in clinically unaffected, adult cats (Legendre, Biggerstaff, Kuritz, 2013, unpublished). Cerna et al. (13) reported PI-associated increases in IFN-α and IFN-γ concentrations in PBMC from SPF kittens and confirmed earlier observations that PI upregulated IL-1β mRNA transcription and induced TLR-dependent TNFα expression *in vitro* (14).

Lee et al. (15) reported that polyprenyl immunostimulant produced measurable innate immune activation through the sphingolipid pathway and shifted the CD4+/CD8+ cell ratio toward CD4+ in various murine tissues. This finding supports the hypothesis that PI may act through an immune-conditioning or priming mechanism. Such upstream regulation could alter early host responses to rechallenge. This interpretation is consistent with the literature, indicating that serum antibody titers do not fully explain protection against FHV-1. PI may therefore influence the host response to rechallenge, reducing the incidence of ocular disease without necessarily increasing VN titers.

We hypothesize that PI may act through an immune-priming mechanism, potentially within a trained-immunity concept (16), to influence early host responses to viral challenge and reduce the likelihood of ocular disease after rechallenge. This possibility is supported only indirectly by the combination of reduced incidence of ocular disease and the absence of detectable differences in serum VN titers, suggesting that any protective effect was not captured by humoral measurements alone. This interpretation is also broadly consistent with prior clinical observations that PI affected survival outcomes (17–19) and inflammatory markers (20) in cats with FIP.

This study was limited by a small sample size and did not include simultaneous testing for cellular innate immunity markers; no PBMC/cytokine/mucosal immune profiling was performed. Further controlled studies addressing the dynamics of immunity markers are needed to determine potential mechanisms by which PI may influence the response to FeHV-1.

## Data Availability

The data supporting the Phase 2 ocular rechallenge outcome are summarized in the article. Additional de-identified Phase 2 clinical outcome data relevant to the ocular rechallenge analysis may be made available by the corresponding author upon reasonable request, subject to applicable regulatory, confidentiality, and record-retention requirements.
